# Structure-Dependent Inhibition of *Stenotrophomonas maltophilia* by Polyphenol and Its Impact on Cell Membrane

**DOI:** 10.3389/fmicb.2019.02646

**Published:** 2019-11-13

**Authors:** Yuxiang Zhang, Jianping Wei, Yue Qiu, Chen Niu, Zihan Song, Yahong Yuan, Tianli Yue

**Affiliations:** ^1^College of Food Science and Engineering, Northwest A&F University, Yangling, China; ^2^Laboratory of Quality & Safety Risk Assessment for Agro-products, Ministry of Agriculture, Yangling, China; ^3^National Engineering Research Center of Agriculture Integration Test, Yangling, China; ^4^College of Food Science and Technology, Northwest University, Xi’an, China

**Keywords:** *Stenotrophomonas maltophilia*, spoilage potential, antibacterial activity, structure-activity relationship, cinnamic acid, membrane damage

## Abstract

As natural occurring antimicrobial substances, phenolic compounds have been used to inhibit various bacteria. *Stenotrophomonas maltophilia* 4–1, a strain isolated from food, exhibited spoilage potential *in vitro* with proteolysis and lipolysis at 25°C. The present study evaluated the antibacterial properties of 13 polyphenols on *S. maltophilia* 4–1, and selected 6 compounds (ferulic acid, *p*-coumaric acid, caffeic acid, chlorogenic acid, (−)-epigallocatechin, and phloretin) for binary combination treatments. The results revealed that antibacterial activities of polyphenols were structure-dependent, and cinnamic acid showed strong inhibitory effects, with a minimum inhibitory concentration (MIC) of 0.125 mg/mL. Importantly, we did not observe any obvious synergistic effects across all binary combinations. The antibacterial mechanism of cinnamic acid was related to membrane damage, caused by the loss of cell membrane integrity and alteration of cell morphology. These findings suggest that cinnamic acid is a promising candidate for the control of spoilage bacteria in food.

## Graphical Abstract

The schematic diagram of the research idea and the experimental process.

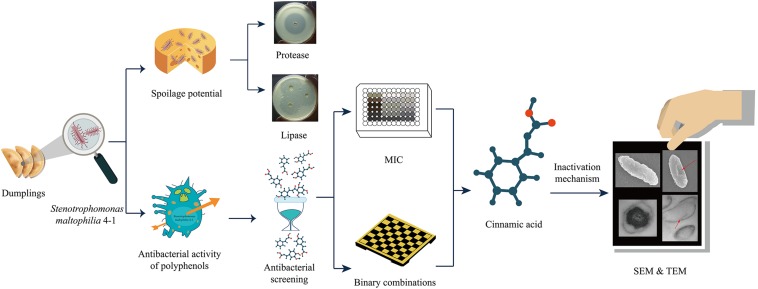

## Introduction

*Stenotrophomonas maltophilia* is an aerobic, non-fermentative, polar flagellate, gram-negative bacillus, which is ubiquitous in the environment and has emerged as an important human opportunistic pathogen. The organism is commonly found in hospitals and can cause pneumonia, urinary tract infections, skin and soft tissue infections and bloodstream infections, especially in immunocompromised patients (Denton and Kerr, 1998). While in the food industry, *S. maltophilia* is also widely distributed. In the raw milk (Munsch-Alatossava and Alatossava, 2006), cod products (Rodrigues et al., 2003), spoiled vegetables (Lee et al., 2013), and even in drinking water (Gomes et al., 2018), the bacterium was present and contributed to the spoilage of food, leading to economic losses and human health threat. Therefore, it is imperative to develop strategies that effectively control and limit the growth and spread of *S. maltophilia*. However, given the multidrug resistance linked to the cryptic efflux pumps and outer membrane proteins (Alonso and Martínez, 1997), *S. maltophilia* is very resistant to conventional antibiotic therapy. Consequently, there is an urgent need to exploit and develop new substances in the hope of inhibiting and killing *S. maltophilia*.

Recently, utilization of polyphenols is becoming increasingly popular as a safe antibacterial strategy. Polyphenols are a class of secondary metabolites with polyphenolic structure, and are produced by higher plants. They are generally divided into flavonoids and non-flavonoids (mainly including phenolic acids) based on their chemical structure (Daglia, 2012). Most phenolic acids can be obtained by the microbial metabolism of some dietary phenolic compounds (such as flavan-3-ols, flavonols, flavones and anthocyanins) (Aura, 2008).

The antimicrobial activity of polyphenol has been proved to be structurally dependent (Cueva et al., 2010; Cushnie and Lamb, 2011), and has been extensively investigated against a wide range of microorganisms. Moreover, flavonoids have received a lot of attention owing to their broad-spectrum antimicrobial activity (Cushnie and Lamb, 2011; Daglia, 2012). Many phenolic acids, such as benzoic acid, cinnamic acid and ferulic acid, have also shown great potential in terms of antibacterial effects (Cueva et al., 2010; Karunanidhi et al., 2013; Shi et al., 2016b). Specifically, polyphenols can directly damage cells to impart their antimicrobial action. Cell wall polypeptides, membrane-bound enzymes, and surface-exposed adhesins are all possible binding targets for phenolic substances. Flavonoids, especially lipophilic flavonoids may also disrupt cytoplasmic membranes (Ahmad et al., 2015).

With respect to *S. maltophilia*, many researchers have reported its sensitivity toward polyphenols. Catechins, such as epicatechin and epigallocatechin-3-gallate, have shown significant bactericidal effects to *S. maltophilia* (Gordon and Wareham, 2010; Betts et al., 2011). Chlorogenic acid has also been proven to be an effective antimicrobial compound for drug-resistant *S. maltophilia* (Karunanidhi et al., 2013). Hence, polyphenols are expected to be promising agents to control the *S. maltophilia*.

In this study, we used *S. maltophilia* 4-1 as research object and estimated its spoilage potential. Then, a comprehensive evaluation of the antibacterial properties of polyphenolics was conducted in 96-well microtiter plates by monitoring optical density (OD) values. Minimum inhibitory concentrations (MICs) of typical polyphenols against *S. maltophilia* were determined. The potential synergistic effects of the binary combinations of polyphenols were also evaluated. In addition, possible antibacterial mechanisms were investigated concentrating on membrane injury. Morphological changes of cell membrane were assessed using field emission scanning electron microscopy (FESEM) and transmission electron microscopy (TEM).

## Materials and Methods

### Bacterial Strain

*Stenotrophomonas maltophilia* 4–1 was isolated from quick-frozen boiled dumpling, and was deposited in the laboratory of Healthy Food Manufacturing Engineering and Food Safety Control at the College of Food Science and Engineering, Northwest A&F University (Yangling, China).

The strain 4–1 was cultured and propagated in nutrient broth (NB; 10 g peptone, 5 g NaCl and 3 g beef extract in 1 L distilled water) or nutrient agar (NA).

### Reagents

Chlorogenic acid (CAS: 327-97-9), neochlorogenic acid (CAS: 906-33-2), *trans*-cinnamic acid (CAS: 140-10-3), ferulic acid (CAS: 1135-24-6), *p*-coumaric acid (CAS: 501-98-4), caffeic acid (CAS: 331-39-5), *p*-hydroxybenzoic acid (CAS: 99-96-7), epicatechin (CAS: 490-46-0), (+)-catechin (CAS: 154-23-4), (−)-epigallocatechin (CAS: 970-74–1), (−)-gallocatechin (CAS: 3371-27-5), phloretin (CAS: 60-82-2) and phlorizin (CAS: 60-81-1) were purchased from Shanghai Yuanye Bio-technology Co., Ltd (Shanghai, China). All phenolic chemicals were analytical standards, with HPLC purity ≥ 98%.

Stock solutions of polyphenols were obtained by dissolving each polyphenol in methanol containing 5% dimethyl sulfoxide (DMSO). Subsequently, each polyphenol was diluted with sterilized NB medium to the appropriate concentration. All solutions were prepared fresh and filter-sterilized (pore size, 0.22 μm) prior to use.

### Spoilage Potential

The pH values of bacterial fermentation broths were determined by a pH meter (Mettler-Toledo, FE28-standard, Switzerland). Before the measurement, bacterial cells were discarded by centrifugation at 5000 rpm for 10 min.

Proteolytic activity of isolates was evaluated using skim milk agar according to previously published protocols (Odeyemi et al., 2018). The tested plates were made of sterilized NA (121°C, 15 min) with 10 g of skim milk (104°C, 15 min) per liter. Every plate consisted of several wells (6 mm in size) containing 100 μL of inoculum or 100 μL of neutral protease solution. After inoculation, the plates were incubated at 4 or 25°C for 10 days. The appearance of clear zones surrounding the colonies indicated the occurrence of proteolytic activity.

As for the assessment of the ability to decompose fat, a series of NA plates supplemented with 10 g/L tributyrin were carefully prepared. Identically, bacterial cultures were inoculated into wells and the plates were kept for 10 days at 4 or 25°C. Lipase was used as a positive control. The difference in lipolytic activity was visualized by the size of the clear decomposition zone.

### Antimicrobial Activity of Polyphenols

#### Preliminary Screening of Polyphenols

All 13 phenolic solutions were used for bacteriostatic screening against *S. maltophilia* 4–1. The bacteriostatic assay was performed following a previous method (Othman et al., 2011) with some modifications. Two negative controls were set up in the experiment, one for the solvent control (CK1) and the other for the NB medium control (CK2). Assays were carried out in 96-well microtiter plates with 250 μL as a reaction volume per well, consisting of 50 μL of the inoculum and 200 μL of NB medium with polyphenols or control solutions. The final concentrations of polyphenols were set at 1 mg/mL, and the initial bacterial concentration was about 10^6^ cells per well. The outer wells in the 96-well plate were left empty to avoid the edge effect. Next, the plates were incubated at 25°C for 48 h and the OD values at 600 nm were measured at fixed time intervals (iMark Microplate Reader, Bio-Rad, Hercules, CA, United States). Empty wells were also used in each experimental series to identify and subtract the noise signal.

#### Minimum Inhibitory Concentrations (MICs) of 7 Polyphenols

The MICs of polyphenols were determined using microdilution method (Othman et al., 2011). After screening, 7 polyphenol compounds (cinnamic acid, ferulic acid, *p*-coumaric acid, caffeic acid, chlorogenic acid, (−)-epigallocatechin, and phloretin) were selected for further study. Operation methods and experimental procedures were the same as section “Preliminary Screening of Polyphenols.” Four final concentration gradients of polyphenol solutions were set as 1, 0.5, 0.25, and 0.125 mg/mL. The OD values at 0 h were subtracted from the OD values after 48 h of incubation, and the obtained data were analyzed to determine the MIC values.

#### Binary Combination Treatments of Polyphenols

The potential synergistic effects of binary combinations of polyphenols were examined by the checkerboard method (Requena et al., 2019). The specific combinations were as follows: 6 different polyphenols (ferulic acid, *p*-coumaric acid, caffeic acid, chlorogenic acid, (−)-epigallocatechin, and phloretin) were combined by binary pairing, and 4 concentrations (1, 0.5, 0.25, and 0.125 mg/mL) of each polyphenol were prepared, then a comprehensive experiment was carried out in a volume ratio of 1:1. In summary, 15 polyphenol combinations were formed, and in each combination there were 16 sets of various treatment concentrations and 2 negative controls (solvent and NB medium) for a total of 270 combinations (Supplementary Table S1). The bacteriostatic method was the same as that described in section “Preliminary Screening of Polyphenols.” The OD values were monitored at 0, 24, 48, and 72 h.

### Evaluation of Bacterial Membrane Integrity

The percentage of cell membrane damage was determined using LIVE/DEAD^®^ BacLight^TM^ Bacterial Viability Kit (Molecular Probes, Invitrogen), according to a previous study (Shi et al., 2016a) and with minor modifications. The re-activated bacterial 4–1 was cultured overnight, and the cells were collected by centrifugation (5000 rpm, 10 min). The pellet was washed twice and resuspended in 2 mL of 0.85% NaCl. To obtain live and dead bacteria, 1 mL of the suspension was separately added to 20 mL of 0.85% NaCl or 20 mL of 70% isopropyl alcohol (chromatographic grade), respectively, and then incubated at 25°C for 1 h (shaking every 15 min to fully react). Afterward, both samples were centrifuged (10,000 rpm, 10 min), rinsed, and resuspended in 0.85% NaCl. The OD_600_ values of the two bacterial solutions were adjusted to 0.5, and a standard group of 5 live bacterial ratios (0, 10, 50, 90, and 100%) was made. Meanwhile, 2 mL of 2X working stain solutions were prepared, including 6 μL of SYTO 9 and 6 μL of PI dye.

Bacterial cells were treated with cinnamic acid at various concentrations (0, MIC/2, MIC, 2MIC and 4MIC) for 30 min at 25°C. Then, the cells were centrifuged (10,000 rpm, 1 min) and resuspended in 0.85% NaCl (OD_600_ = 0.5). Aliquots of 100 μL of the bacterial solutions or standard samples and 100 μL of the 2X stain solutions were pipetted in three parallels into black 96-well microtiter and mixed thoroughly by pipetting up and down several times in the dark. The fluorescence was measured with a multi-function microplate detector (Spectra Max M2, Molecular Devices, United States). The excitation/emission spectral wavelengths of the dyes were 485/530 nm (em1; green) for SYTO 9 and 485/630 nm (em2; red) for PI.

The percentage of live cells was expressed as the ratio of integrated green fluorescence to integrated red fluorescence (Ratio _G/R_):

$${\text{Ratio}}_{\text{G}/\text{R}}=\frac{F_{c\,e\,l\,l,e\,m\,1}}{F_{c\,e\,l\,l,e\,m\,2}}$$

where F is the fluorescence, G is the green channel and R is the red channel.

### Morphological Assessment of Bacterial Structure

Morphological changes of bacteria were observed by field emission scanning electron microscopy (FESEM) (Zhang et al., 2019) and transmission electron microscopy (TEM) (Ren et al., 2019). Bacterial cells were treated with cinnamic acid at 0 and MIC. After incubation for 2 h at 25°C, cells were harvested by centrifugation (5000 rpm, 10 min) and washed twice with PBS (pH 7.0).

For assessment by FESEM, a coverslip (<7.0 mm) was placed in a high concentration of bacterial solution for sample attachment. Subsequently, cells were fixed with 2.5% (v/v) glutaraldehyde for 2 h, rinsed three times with PBS, followed by dehydration with an ethanol gradient (30, 50, 70, 80, 90, and 100%). Afterward, the samples were dried by CO_2_ critical point drying for 4 h; then fixed on sample support, and sputter-coated with gold under vacuum. The observations were carried out using a Hitachi S-4800 FESEM (Hitachi Limited, Japan).

For TEM analysis, the washed cell pellets were first fixed with glutaraldehyde and rinsed, and then fixed again with 1% osmium tetroxide (OsO_4_) and rinsed thoroughly. Next, the samples were dehydrated in a series of ethanol solutions and infiltrated with LR-White resin (London Resin Company, Reading, United Kingdom). Then the samples were embedded with pure LR-White resin and incubated for 48 h at 60°C. After obtaining ultrathin sections and double staining, all sections were examined under a JEM-1230 TEM (JEOL, Tokyo, Japan).

### Data Analysis

All experiments were performed in triplicate, and each biological replicate included two technical replicates for a total of 6 runs. One-way analysis of variance and Duncan’s multiple-range test were carried out with SPSS 22.0 for Windows (SPSS Inc., Chicago, IL, United States).

In the analysis of binary combinations, the results of three replicates were averaged to obtain the final average OD value, which was subsequently used to generate heatmap in R software^1^.

## Results

### Spoilage Potential

Spoilage potential assessment of *S. maltophilia* 4–1 revealed that the bacteria were found to produce high levels of proteases and lipases at 25°C (Table 1 and Supplementary Figure S1). Particularly, the lipolytic activity was significantly higher (*p* < 0.05) compared to the positive control (lipase) at 25°C. When the temperature dropped to 4°C, the strain exhibited no proteolytic activity, but it still maintained a slight lipolytic activity. Moreover, the pH values of the fermentation supernatant were affected by temperature.

**TABLE 1:** The spoilage potential of *S. maltophilia* 4–1 at 4 and 25°C.

**Temperature**	**pH of fermentation broth**		**Proteolytic activity (mm)**		**Lipolytic activity (mm)**	
	***S. maltophilia* 4–1**	**NB medium**	***S. maltophilia* 4–1**	**Neutral protease**	***S. maltophilia* 4–1**	**Lipase**
4°C	6.71 ± 0.01^by^	6.88 ± 0.02^ax^	0 ± 0^by^	45.63 ± 0.27^ax^	15.12 ± 0.86^by^	33.43 ± 2.05^ax^
25°C	7.62 ± 0.05^ax^	6.92 ± 0.04^ay^	38.16 ± 0.87^ay^	45.13 ± 0.54^ax^	42.35 ± 1.21^ax^	33.72 ± 0.87^ay^

*Values are expressed as the mean ± standard deviation (*n* = 3). NB medium, neutral protease, and lipase were used as controls. ^*ab*^Mean values in the same column with different superscripts differ significantly (*p* < 0.05) by Duncan’s test. ^*xy*^Mean values in the same row from three separate experiments (4–1 *vs*. control) with different superscripts differ significantly (*p* < 0.05) by Duncan’s test.*

### Antibacterial Activity of Polyphenols

#### Preliminary Screening

**FIGURE 1:**
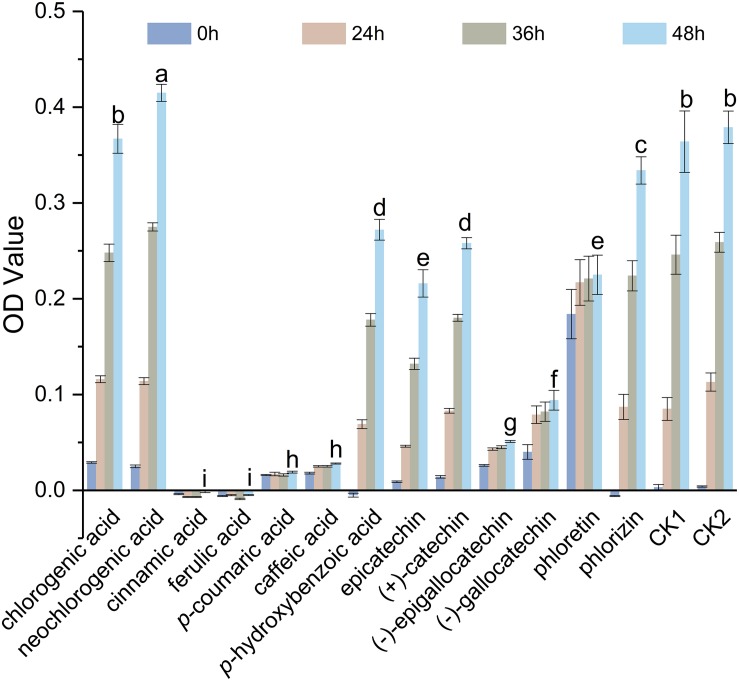
Antibacterial activities of polyphenols against *S. maltophilia* 4–1. CK1, solvent control; CK2, NB medium control. Bars with different letters differ significantly (*p* < 0.05) by Duncan’s test. All experiments were performed in triplicate.

Polyphenols examined in this study showed significant inhibitory effects against *S. maltophilia* 4–1, except for chlorogenic acid and neochlorogenic acid (Figure 1). Cinnamic acid, ferulic acid, *p*-coumaric acid, and caffeic acid exhibited strong antibacterial effects, followed by (−)-epigallocatechin and (−)-gallocatechin. In particular, the background value (at 0 h) of phloretin was high, so its antibacterial effect required additional verification.

#### MICs of Seven Polyphenols

When *S. maltophilia* 4–1 was treated with 7 selected polyphenols, there was a significant inhibition with varying degrees at 4 examined concentrations (Supplementary Figure S2). As shown in Table 2, cinnamic acid exhibited the strongest antibacterial effect with MIC value of 0.125 mg/mL. Concentration of 0.0625 mg/mL was not sufficient to completely inhibit bacterial growth (not shown). Moreover, phloretin was identified as another potent polyphenol with MIC value of 0.25 mg/mL. Finally, the MIC values of ferulic acid, *p*-coumaric acid, caffeic acid, and (−)-epigallocatechin were all 1 mg/mL. Chlorogenic acid could not completely inhibit the growth of bacteria at the four tested concentrations, and its MIC value against *S. maltophilia* 4–1 was greater than 1 mg/mL.

**TABLE 2:** Minimum inhibitory concentrations (MICs) of seven phenolic compounds against *S. maltophilia* 4–1.

**Compounds**	**MIC (mg/mL)**
Cinnamic acid	0.125
Phloretin	0.25
Ferulic acid	1
*p*-Coumaric acid	1
Caffeic acid	1
(−)-Epigallocatechin	1
Chlorogenic acid	>1

#### Binary Combination Treatments

**FIGURE 2:**
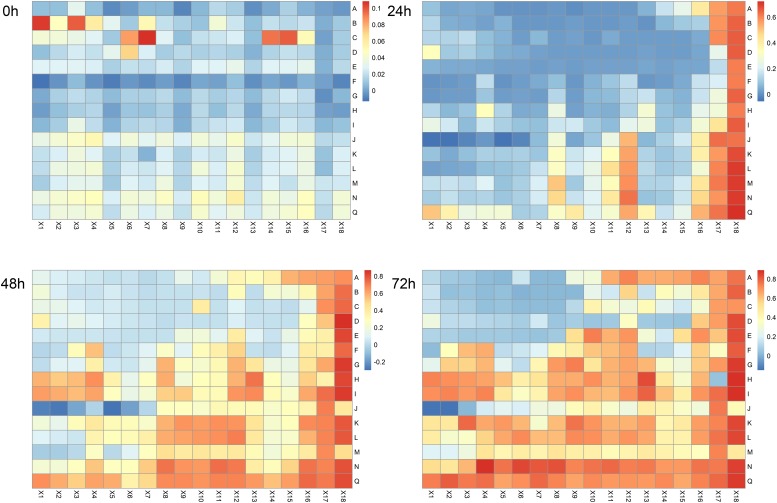
Heatmap of antibacterial effects of binary combination treatments at 0, 24, 48, and 72 h. Bars shown on the right of each map represent OD values. Blue and red indicate low and high levels of OD values, respectively. A, phloretin – ferulic acid; B, phloretin – *p*-coumaric acid; C, phloretin – caffeic acid; D, phloretin – (–)-epigallocatechin; E, phloretin – chlorogenic acid; F, ferulic acid – *p*-coumaric acid; G, ferulic acid – caffeic acid; H, ferulic acid – (–)-epigallocatechin; I, ferulic acid – chlorogenic acid; J, *p*-coumaric acid – caffeic acid; K, *p*-coumaric acid – (–)-epigallocatechin; L, *p*-coumaric acid – chlorogenic acid; M, caffeic acid – (–)-epigallocatechin; N, caffeic acid – chlorogenic acid; Q, (–)-epigallocatechin – chlorogenic acid; X1, 1 mg/mL and 1 mg/mL; X2, 1 mg/mL and 0.5 mg/mL; X3, 1 mg/mL and 0.25 mg/mL; X4, 1 mg/mL and 0.125 mg/mL; X5, 0.5 mg/mL and 1 mg/mL; X6, 0.5 mg/mL and 0.5 mg/mL; X7, 0.5 mg/mL and 0.25 mg/mL; X8, 0.5 mg/mL and 0.125 mg/mL; X9, 0.25 mg/mL and 1 mg/mL; X10, 0.25 mg/mL and 0.5 mg/mL; X11, 0.25 mg/mL and 0.25 mg/mL; X12, 0.25 mg/mL and 0.125 mg/mL; X13, 0.125 mg/mL and 1 mg/mL; X14, 0.125 mg/mL and 0.5 mg/mL; X15, 0.125 mg/mL and 0.25 mg/mL; X16, 0.125 mg/mL and 0.125 mg/mL; X17, solvent control; X18, NB medium control. The intersections of rows and columns represent binary combination (A–Q) with corresponding concentrations (X1–X16).

Data expressed as heatmaps (Figure 2) indicated that the antibacterial effects of polyphenol combinations were gradually increased over time. Specifically, at 72 h we observed strong bacteriostatic effects mainly concentrating in the top left corner (AX1-EX8) of the heatmap, which represented the combinations of phloretin and the other 5 polyphenols (Supplementary Table S1). In addition, the JX1–JX3 part (combination of *p*-coumaric acid and caffeic acid) and the row D [combination of phloretin and (−)-epigallocatechin] also shown strong bacteriostatic effects. Overall, these data suggested that bacteriostatic phenomenon principally occurred at high concentrations of polyphenols with phloretin playing an important role.

### Assessment of Cell Membrane Injury by Fluorescence

**FIGURE 3:**
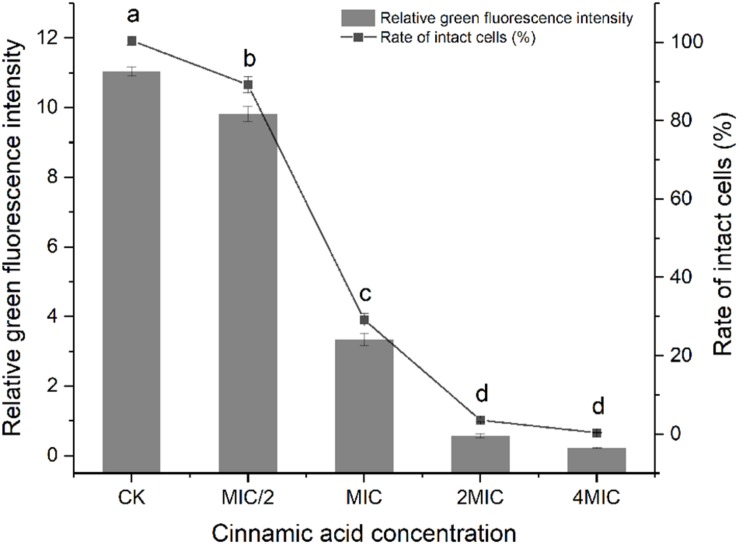
Effect of cinnamic acid at different concentrations on the membrane integrity of *S. maltophilia* 4–1. CK represents the group without citral. Bars with different letters differ significantly (*p* < 0.05) by Duncan’s test. All experiments were performed in triplicate.

There was a good linearity between the ratio of green fluorescence intensity and the percent of viable bacteria (*R*^2^ = 0.98). Cells of *S. maltophilia* 4–1 in the control group (CK) exhibited good growth. After treatment with cinnamic acid, significant decreases (*p* < 0.05) in living cells were observed (Figure 3). Membrane-damaged cells in MIC/2 group were 11%. Cinnamic acid at MIC caused a 71% reduction of living cells, while 2MIC and 4MIC almost caused the total death of *S. maltophilia* 4–1.

### Microscopic Observation

**FIGURE 4:**
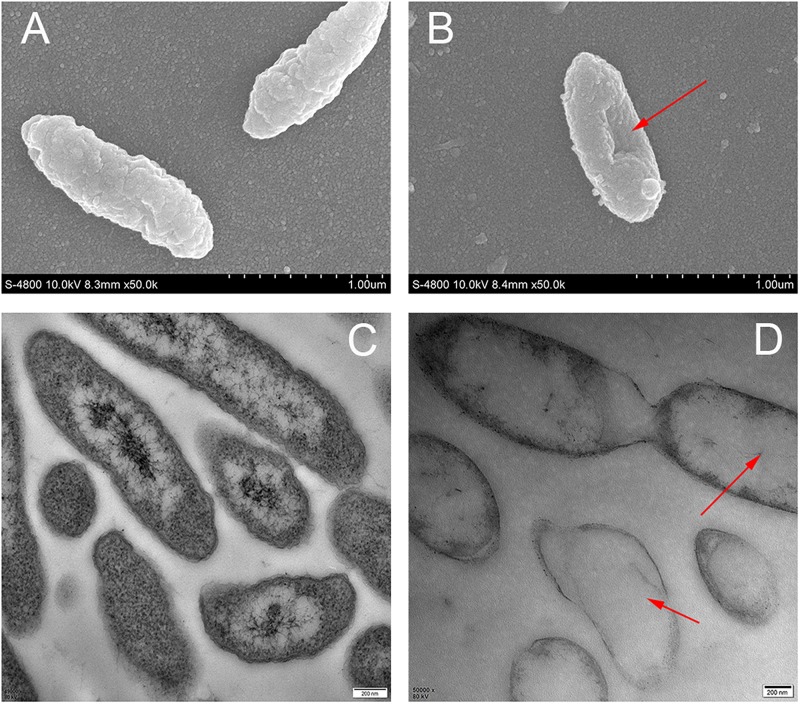
Images of scanning electron microscopy **(A,B)** and transmission electron microscopy **(C,D)** of *S. maltophilia* 4–1 before **(A,C)** and after **(B,D)** cinnamic acid treatment.

Analysis by FESEM demonstrated that the changes in cell membrane occurred after treatment with cinnamic acid. Untreated cells had intact cell membranes (Figure 4A), while bacterial cells treated with cinnamic acid was depressed and shrunk (showed by red arrows in Figure 4B). In the TEM images, the cytoplasm of untreated cells was uniform and dense (Figure 4C), while in treated cells, we observed the leakage of cellular contents and the damage of cytoplasm (showed by red arrows in Figure 4D). It could also be seen that the cell membrane had broken.

## Discussion

### Spoilage Potential

*Stenotrophomonas maltophilia*, widely considered to be an important nosocomial pathogen found in bedridden and immunocompromised patients (Denton and Kerr, 1998), has been extensively studied in clinical and epidemiological fields. However, the *S. maltophilia* 4–1 used in this experiment was derived from food and obtained from 4°C; therefore, we evaluated its spoilage potential at both low and normal temperatures. Based on the literature evidence, *S. maltophilia* could produce high levels of proteases (Ben-Gigirey et al., 2000; Ercolini et al., 2009), but its production of lipases depended on the strain (Ben-Gigirey et al., 2000; Cleto et al., 2012). In our study, superior secretion of extracellular lipase and protease was observed at 25°C in *S. maltophilia* 4–1 (Table 1 and Supplementary Figure S1). Importantly, the lipolytic and proteolytic activities of 4–1 were greatly affected by temperature. Although some *S. maltophilia* exhibited a certain cold tolerance, *S. maltophilia* 4–1 in this study preferred to grow and metabolize in a mildly warm environment (25°C). In our study, the bacteria grew slowly with low vitality, and only showed a little lipolytic activity at 4°C, which implied that *S. maltophilia* 4–1 was most likely mesophilic (Ben-Gigirey et al., 2000).

### Structure-Dependent Antibacterial Activity

Since *S. maltophilia* are both pathogenic and spoilage-associated, it is necessary to take appropriate measures to control them. Polyphenols were used as antibacterial substances in view of the multidrug resistance of *S. maltophilia*. Considering chemical structure, the polyphenols are composed of a wide variety of molecules with polyphenol structure and are generally divided into phenolic acids and flavonoids (Spanos and Wrolstad, 1992; Daglia, 2012). Most of these two groups exhibit antimicrobial activity and have the ability to suppress some microbial virulence factors. Especially, flavonoids have received more attention due to their broad spectrum and higher antimicrobial activity in comparison with phenolic acids (Cushnie and Lamb, 2011; Daglia, 2012; Kumar and Pandey, 2013).

#### Flavonoids

**FIGURE 5:**
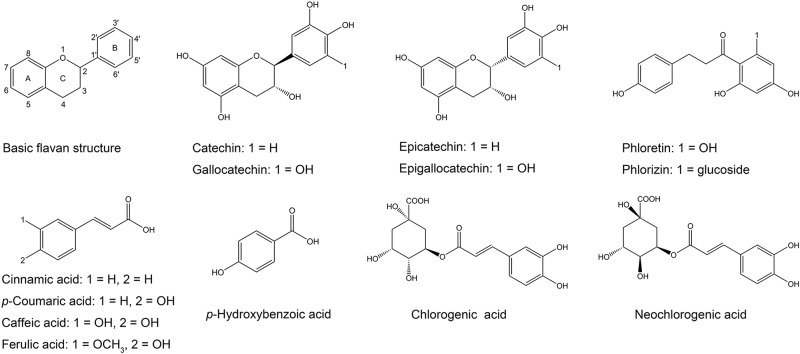
Chemical structures of phenolic compounds.

Flavonoids share a basic skeleton of diphenyl propane (C6–C3–C6), as depicted in Figure 5, consisting of two benzene rings (ring A and B) linked via a heterocyclic pyran ring (ring C) (Daglia, 2012; Cheynier and Halbwirth, 2017). Based on the number of substituents, the degree of unsaturation, and the oxidation state of the three-carbon segment, flavonoids can be subdivided into many subclasses (Spanos and Wrolstad, 1992). Flavan-3-ols, also known as catechins, are common and widely occurring flavonoids. They are the main active ingredient of tea polyphenols and are responsible for the antimicrobial activity (Almajano et al., 2008).

The tolerance of bacteria to polyphenols depends on both the bacterial species and the polyphenol structure (Almajano et al., 2008). Many researchers have studied the bacteriostatic effect of catechins on *S. maltophilia*. Epigallocatechin-3-gallate (EGCG) has been shown to exhibit antimicrobial effects against several bacterial pathogens, including *S. maltophilia* (Navarro-Martinez et al., 2005; Gordon and Wareham, 2010). In the present study, catechins also exhibited good antibacterial effects against *S. maltophilia* 4–1, particularly (−)-epigallocatechin (EGC) and (−)-gallocatechin (GC), while the bacteriostatic effects of (+)-catechin and epicatechin were overall weak. This may be likely explained by the structure of phenolic substances (Figure 5). Hydroxylation at position 5 and 7 of the ring A has been reported to be important for bacteriostatic action (Cushnie and Lamb, 2011). Moreover, the ring B of flavonoids may inhibit the synthesis of DNA and RNA in bacteria by inserting or forming hydrogen bonds with the stacking of nucleic acid bases (Kumar and Pandey, 2013). Therefore, catechins exhibit a certain level of antibacterial activity. The isomeric catechin and epicatechin differ in the *cis*-*trans* conformation at position 2 and 3 (Figure 5). Catechins have the number 2 and 3 group in *trans* configuration, while there is *cis* configuration in epicatechin. The same conformational difference is also present in EGC and GC isomers. Results from this study suggest that the *cis*-form of catechins is more conductive to bacteriostatic activity compared to the *trans*-form. Compared to catechin and epicatechin, GC and EGC both have a hydroxyl substitution at the 5′ position in ring B (Figure 5), which significantly enhanced their antibacterial effect. This is consistent with previous studies documenting that polyphenols with pyrogallol groups showed stronger activity than those containing the catechol group (Taguri et al., 2006).

Phloretin belongs to the class of dihydrochalcone compounds (Figure 5), and its major derivative is phlorizin. Our results indicated that the antimicrobial activity of phloretin was better than that of phlorizin, but its solubility was weak. Solubility plays an important role in the therapeutic efficacy and applications of compounds (Kumar and Pandey, 2013). Despite the fact that the effect of phloretin was good, we abandoned this polyphenol in subsequent experiments. Moreover, the addition of glucoside significantly reduced the antibacterial activity of phloretin (Zhang et al., 2016).

#### Phenolic Acids

Although phenolic acids generally show weaker antimicrobial activity in comparison with flavonoids, their activities are still worth discussing. Phenolic acids include the cinnamic acids (C6–C3) and the benzoic acids (C7) (Spanos and Wrolstad, 1992). In this study, *p*-hydroxybenzoic acid was selected to evaluate its antibacterial activity and it could inhibit the growth of 4–1 to some extent (Figure 1). By reports, a hydroxyl substituent in position 4 (*para*-position) of benzoic acids is linked with an improved antimicrobial activity (Cueva et al., 2010). To our surprise, cinnamic acids displayed very strong antibacterial activities against 4–1, which was not in agreement with the previous study (Zheng et al., 2013). Cinnamic acids are one of the major classes of phenolic compounds found in nature, including common caffeic acid, *p*-coumaric acid and ferulic acid (Herrmann, 1989). They showed antibacterial activity against both gram-positive and gram-negative bacteria. Moreover, the reported MIC values of cinnamic acids vary greatly across different species and strains (Wen et al., 2003; Saavedra et al., 2010; Guzman, 2014). A study by Guzman (2014) summarized that *p*-coumaric acid was a more potential inhibitor against most bacteria compared to cinnamic acid, and caffeic acid and ferulic acid were more inclined to inhibit gram-positive bacteria. Nevertheless, in the present study, cinnamic acid had the strongest bacteriostatic activity with a MIC value of 0.125 mg/mL, while the MIC values of the other three cinnamic acid derivatives on *S. maltophilia* were 1 mg/mL. We posit that the benzene ring substitution caused a reduction of antibacterial activity of cinnamic acid (Figure 5). The cinnamic skeleton is regarded as an interesting scaffold for developing novel antimicrobial agents, which have appeared in many active chemical molecules and therapeutic drugs (Guzman, 2014). In addition, cinnamic acid can exist both in *cis* and *trans* forms, while the *trans* configuration is more predominant in nature due to its stability and availability (Herrmann, 1989). The *cis*-cinnamic acid and *trans*-cinnamic acid can be transformed from each other, and several studies have even shown that *cis*-cinnamic acid exerts a higher physiological activity than *trans*-cinnamic acid in many aspects (Spanos and Wrolstad, 1992; Chen et al., 2011).

Cinnamic acids occur naturally in combination with other compounds, usually in the form of esters (Spanos and Wrolstad, 1992). Chlorogenic acid (5-*O*-caffeoylquinic acid) and neochlorogenic acid (3-*O*-caffeoylquinic acid) (Figure 5), which are formed by the condensation of caffeic acid with quinic acid, are probably the most abundant soluble hydroxycinnamic acid derivatives (Nakatani et al., 2000). Chlorogenic acid showed no activity against gram-positive bacteria (Saavedra et al., 2010), while a previous research (Karunanidhi et al., 2013) reported that chlorogenic acid has promising *in vitro* antibacterial activity against *S. maltophilia* with MICs ranging from 8 to 16 μg/mL. But in our study, both chlorogenic acid and neochlorogenic acid had almost no bacteriostatic effect.

#### Binary Combinations

The combination of multiple substances sometimes frequently leads to an enhanced effect. Several studies have attempted to understand the synergistic effects among antibacterial agents, focusing on the synergy between antibiotics and phenolics (Saavedra et al., 2010), as well as the combination of polyphenols (Betts et al., 2011). Based on the screening results, six representative polyphenols were selected for MIC determination and binary treatment experiments. Cinnamic acid with the strongest antibacterial ability was used as a control. In all binary combinations, it was a pity that no strong synergy was observed. The antibacterial effect of cinnamic acid alone was better compared to all binary treatment combinations. Therefore, the inhibition of *S. maltophilia* 4–1 by cinnamic acid was further examined to elucidate its mechanism related to membrane damage.

### Cell Membrane Damage

Due to the limited research on *S. maltophilia*, little information is available on its mechanism of inactivation action. In this study, we investigated the inhibitory effect of cinnamic acid against *S. maltophilia*, and determined the antibacterial mode of action by measuring the cell membrane integrity and cell morphology.

The commercially available bacterial viability kit provides a two-color fluorescent assay that is very useful to study a diverse array of bacterial genera. The kit contains two stains with different spectral characteristics and ability to penetrate healthy bacterial cells. Green fluorescent SYTO9 dye generally labels all cells and it is used for assessing total cell counts, whereas red fluorescent PI dye only enters bacteria with damaged cytoplasmic membranes (Berney et al., 2007). When two stains overlap, PI can cause a reduction in the green fluorescence of SYTO9. Since this commercial kit became available (Boulos et al., 1999), it continues to gain increasing popularity among researchers in various fields. A study by Shi et al. (2016b) utilized SYTO9 nucleic acid staining to estimate the integrity of cell membrane, identifying the bactericidal mechanism of ferulic acid on *Cronobacter sakazakii* strains. Our results also displayed a significant negative correlation between the concentration of cinnamic acid and the ratio of green fluorescence, which confirmed that as the concentration of cinnamic acid shifted from 0 to 4MIC, the number of cells with damaged membranes significantly increased (Figure 3). The cell membrane is an active structure that maintains optimal internal conditions for the metabolism and energy transduction. It acts as a fundamental barrier between the cytoplasm and the extracellular medium (Sanchez et al., 2010). Once this barrier was broken, the bacterial cells therewith lose their activity.

Electron microscopy is a powerful tool for researchers to better understand the effects of stressors on bacterial cells (Chen et al., 2017). FESEM and TEM were carried out to directly observe the physical and morphological alterations in bacterial cell structure. The cinnamic acid revealed its inhibitory effects as confirmed by the severe morphological changes on the cell and the thorough dissolution of intracellular contents of the tested *S. maltophilia*. Similar morphological alterations have also been observed in various kinds of tested bacteria when treated with different phenolic acid or essential oils (Bajpai et al., 2013; Shi et al., 2016b; Chen et al., 2017). However, there are a few reports on the microscopic morphology of *S. maltophilia* cells. In a literature focusing on its biofilm formation, we learned antibiotics could induce *S. maltophilia* cells lysis, DNA condensation forming apoptotic bodies, and degradation of glycocalyx, thereby suppressing biofilm formation, reducing drug resistance, and accelerating cell death (Di Bonaventura et al., 2003).

Deformation of the physical structure of the cell would cause swelling and instability of the membrane, even a relatively slight change to the structural integrity could adversely affect cell metabolism and lead to cell death (Bajpai et al., 2013; Chen et al., 2017). The bacteriostatic mechanism of phenolic acids is to cause irreversible changes in the cell membrane by altering hydrophobicity and local rupture or pore formation in the cell membrane (Chen et al., 2017). These phenolic compounds primarily target the cytoplasmic membrane via accumulation of hydroxyl group in the lipid bilayer, which disrupt the interaction of lipid protein, increase the permeability of cell membrane, give rise to the leakage of cellular contents, disrupt the proton-motive force and electron influx, and ultimately destroy cell integrity (Shi et al., 2016a).

Many studies have investigated the antibacterial mechanism of natural products from the perspective of gene expressions. Chueca et al. (2017) have studied the mechanism of bacterial inactivation using DNA microarrays, and have concluded that carvacrol and citral caused membrane damage in *Escherichia coli*. Similarly, Lin et al. (2017) have also analyzed the antibacterial mechanism of cinnamaldehyde against *E. coli* by microarray analysis and verified the expressions of related genes by RT-PCR. The results indicated that flagellin and cell membrane may be the preliminary antibacterial mechanism of cinnamaldehyde. Pterostilbene, a natural polyphenol, has been proven to have good inhibitory effects on *E. coli* and *Staphylococcus aureus* (Ren et al., 2019). It could damage cell membranes, cause leakage of nucleic acids and proteins, and produce reactive oxygen species. Changes in the expression levels of genes were confirmed by qRT-PCR, and it was found that the genes related to oxidative stress were up-regulated, and the genes related to cell wall synthesis were down-regulated after pterostilbene treatment. The essential oil from Huyou plant could effectively destroy the cell membrane and biofilm of *Listeria monocytogenes*, and RNA sequencing results showed that differentially expressed genes were enriched in pathways such as protein export, bacterial secretion systems, ABC transporter, and quorum sensing (Guo et al., 2019). A transcriptomic analysis revealed the upregulation of related stress resistance genes in essential oil-adapted *E. coli* cells (treated with antibacterial agents at one-half MIC), and these cells have shown enhanced resistance against subsequent lethal essential oil (Yuan et al., 2018). All these findings have demonstrated that natural products can cause changes in the expression levels of genes in bacterial cells, especially those associated with membranes. However, there were few studies on inactivation of *S. maltophilia* induced by polyphenol, which provided a good entry point for our future research. It may be necessary and valuable to further explore the antibacterial mechanism of cinnamic acid on *S. maltophilia* 4-1 at molecular and gene levels.

## Conclusion

*Stenotrophomonas maltophilia* is an emerging opportunistic pathogen linked with an increasing number of clinical syndromes. Abundant reports have focused on its infectivity and biofilm-related multidrug resistance. As a food-acquired strain, *S. maltophilia* 4–1 exhibited strong spoilage capacity at 25°C with high production of extracellular lipase and protease. Natural antimicrobial agents of plant origin have great potential in food industry due to their bacteriostatic ability and safety satisfying consumers’ pursuit of additive-free, fresher and more natural-tasting food. The present study was designed to evaluate the antibacterial activities of phenolic substances, and to systematically uncover the relationship between structure and activity at a functional group level. This work provides guidance and theoretical basis for the control of spoilage bacteria via polyphenols. Meanwhile, the inhibition effects of binary combination of phenolic compounds were investigated through the checkerboard method. However, none of the binary combinations was superior to the cinnamic acid alone in terms of bacteriostatic effects. The main mechanism of cinnamic acid against *S. maltophilia* 4–1 was further verified to be linked with the loss of structural integrity and the disruption of the actual membrane. Cinnamic acid was expected to be a promising agent for inactivating *S. maltophilia* in various foods. Although our preliminary results are encouraging, additional *in vivo* studies are needed to examine its inhibitory activities in food matrixes and to better understand its effects on the sensory characteristic of food products, ensuring that cinnamic acid becomes available for commercial application.

## Data Availability Statement

All datasets generated for this study are included in the article/Supplementary Material.

## Author Contributions

YZ, JW, and TY conceived and designed the experiments. YZ and JW performed the experiments and analyzed the data. YZ, JW, and YQ searched the literature and wrote sections of the manuscript. CN, ZS, and YY organized the framework and revised the manuscript. All authors contributed to manuscript revision, read, and approved the submitted version.

## Conflict of Interest

The authors declare that the research was conducted in the absence of any commercial or financial relationships that could be construed as a potential conflict of interest.

## Footnotes

1 1http://www.r-project.org/
