# Supplementary material for: Structure-Dependent Inhibition of Stenotrophomonas maltophilia by Polyphenol and Its Impact on Cell Membrane
Source: Front Microbiol. 2019 Nov 13;10:2646. doi: 10.3389/fmicb.2019.02646 (PMC6863799; doi:10.3389/fmicb.2019.02646)
Supplement: Supplementary file 2 [file Table_2.DOCX]

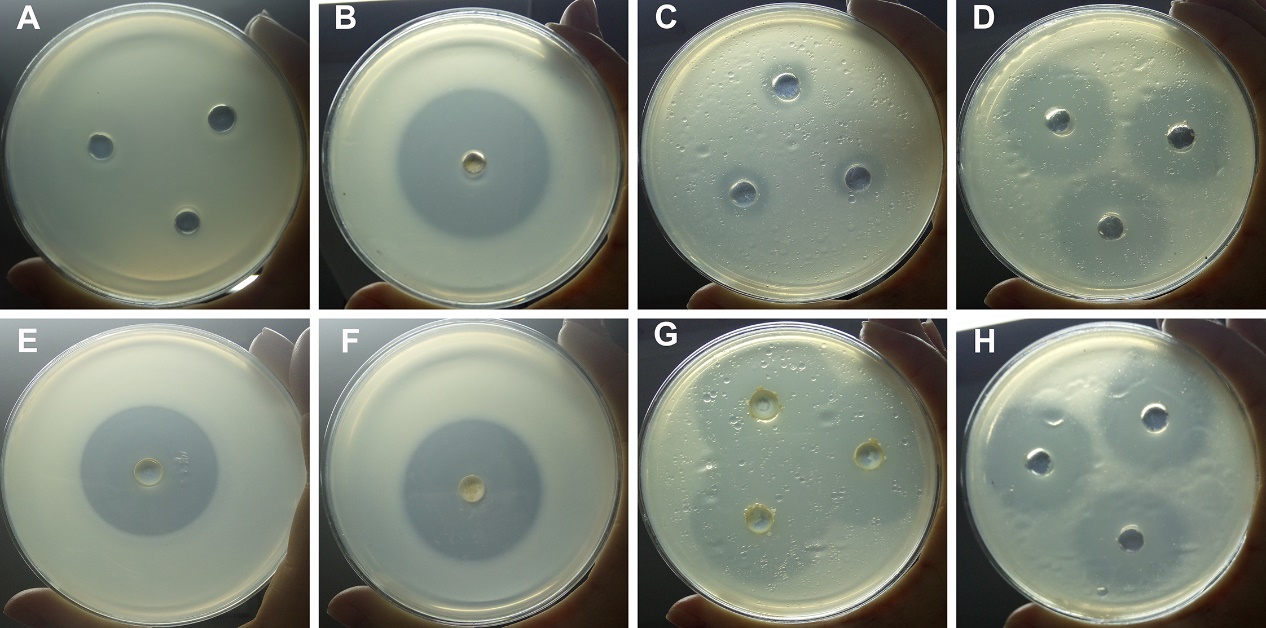


Figure S1. The proteolytic and lipolytic activity of *S. maltophilia* 4–1 at 4°C and 25°C. (A) and (B) show proteolysis at 4°C, induced by 4–1 and neutral protease, respectively; (C) and (D) show lipolysis at 4°C, induced by 4–1 and lipase, respectively; (E) and (F) show proteolysis at 25°C, induced by 4–1 and neutral protease, respectively; (G) and (H) show lipolysis at 25°C, induced by 4–1 and lipase, respectively.





Figure S2. Antibacterial activities of seven polyphenols against *S. maltophilia* 4–1. CK1 = solvent control, CK2 = NB medium control.
